# Immune sensitization to methylene diphenyl diisocyanate (MDI) resulting from skin exposure: albumin as a carrier protein connecting skin exposure to subsequent respiratory responses

**DOI:** 10.1186/1745-6673-6-6

**Published:** 2011-03-17

**Authors:** Adam V Wisnewski, Lan Xu, Eve Robinson, Jian Liu, Carrie A Redlich, Christina A Herrick

**Affiliations:** 1 Department of Internal Medicine; Yale University School of Medicine; 300 Cedar Street; New Haven, CT; 06510, USA; 2 Department of Dermatology; Yale University School of Medicine; 300 Cedar Street; New Haven, CT; 06510, USA

## Abstract

**Background:**

Methylene diphenyl diisocyanate (MDI), a reactive chemical used for commercial polyurethane production, is a well-recognized cause of occupational asthma. The major focus of disease prevention efforts to date has been respiratory tract exposure; however, skin exposure may also be an important route for inducing immune sensitization, which may promote subsequent airway inflammatory responses. We developed a murine model to investigate pathogenic mechanisms by which MDI skin exposure might promote subsequent immune responses, including respiratory tract inflammation.

**Methods:**

Mice exposed via the skin to varying doses (0.1-10% w/v) of MDI diluted in acetone/olive oil were subsequently evaluated for MDI immune sensitization. Serum levels of MDI-specific IgG and IgE were measured by enzyme-linked immunosorbant assay (ELISA), while respiratory tract inflammation, induced by intranasal delivery of MDI-mouse albumin conjugates, was evaluated based on bronchoalveolar lavage (BAL). Autologous serum IgG from "skin only" exposed mice was used to detect and guide the purification/identification of skin proteins antigenically modified by MDI exposure in vivo.

**Results:**

Skin exposure to MDI resulted in specific antibody production and promoted subsequent respiratory tract inflammation in animals challenged intranasally with MDI-mouse albumin conjugates. The degree of (secondary) respiratory tract inflammation and eosinophilia depended upon the (primary) skin exposure dose, and was maximal in mice exposed to 1% MDI, but paradoxically limited in mice receiving 10-fold higher doses (e.g. 10% MDI). The major antigenically-modified protein at the local MDI skin exposure site was identified as albumin, and demonstrated biophysical changes consistent with MDI conjugation.

**Conclusions:**

MDI skin exposure can induce MDI-specific immune sensitivity and promote subsequent respiratory tract inflammatory responses and thus, may play an important role in MDI asthma pathogenesis. MDI conjugation and antigenic modification of albumin at local (skin/respiratory tract) exposure sites may represent the common antigenic link connecting skin exposure to subsequent respiratory tract inflammation.

## Background

Isocyanates, the reactive chemicals used in the production of polyurethane foams, coatings, and adhesives remain a leading cause of occupational asthma world-wide, despite substantial efforts at disease prevention [1]. MDI has become the most commonly used isocyanate for multiple reasons, including its relatively low volatility at room temperature, which has been presumed to make it "safer" than other major isocyanates, e.g. hexamethylene and toluene diisocyanate (HDI and TDI respectively) [2,3]. However, respirable forms of MDI are inherent to its common applications, which often involve heating and/or spraying the chemical, thus creating vapor and aerosols. The number of people at risk from MDI exposure continues to increase with increasing demand for polyurethane containing products; for example, "environmentally-friendly" or "green" construction using MDI-based spray-foam insulation made with soybean (vs. petroleum)-derived polyols [2,4,5]. A better understanding of MDI asthma pathogenesis is central to multiple approaches toward protecting the health of occupationally exposed individuals, including hygiene, engineering controls, personal protective equipment, exposure/disease surveillance and treatment [6-9].

Despite decades of research, the pathogenesis of MDI, and other isocyanate (TDI, HDI)-induced asthma remains unclear; however, contemporary theories suggest one important step involves the chemical's reactivity with "self" proteins in the respiratory tract, causing antigenic changes in their structure/conformation, which trigger an immune response [10,11]. The self-proteins crucial to this process remain incompletely defined, however in animal models, the major target for isocyanate in the airways has been identified as albumin, by multiple investigators using several distinct approaches (immunochemical, radiotracing) [12-15]. Albumin has also been found conjugated with isocyanate in vivo in occupationally exposed humans, and is the only known "carrier" protein for human antibody recognition and binding (e.g. IgE/IgG from exposed individuals specifically bind to isocyanate conjugates with human albumin, but not other proteins) [16]. Furthermore, in animal models of TDI and HDI asthma, albumin conjugates have been shown to induce asthma-like airway inflammation and/or physiologic responses in previously (isocyanate) sensitized animals [17-22]. Thus, while the pathogenesis of MDI (and other isocyanate-induced) asthma remains unclear, previous studies support an important role for chemical conjugation with albumin present in the airways.

Given the airway localization of inflammation in isocyanate asthma patients, inhalation was originally assumed to be the primary exposure route responsible for the immune activation associated with exposure. However, evidence continues to increase in support of an alternative hypothesis; that skin exposure is equally (if not more) effective for isocyanate immune sensitization. Skin exposure to isocyanates is relatively common during polyurethane production (likely more common than airway exposure for "low volatility" isocyanates such as MDI) and thus could play a major role in sensitizing workers, despite appropriate respiratory tract protection, and without "warning" (methods for monitoring skin exposure remain poorly developed, and skin reactions are rare). Once immune sensitization to isocyanate occurs, extremely low airborne levels (below OSHA established permissible exposure levels) can trigger asthmatic reactions [23,24]. Thus, while research, practice and regulation have focused almost exclusively on understanding and preventing inhalation exposures [6,25-27], skin exposure may be an equally critical, yet, under-recognized target for isocyanate asthma prevention [6,8,28,29].

In this study, we developed a murine model to investigate the capacity of MDI skin exposure to induce systemic immune sensitization, and to identify key "MDI antigens" in this process. The investigation builds upon previous studies in guinea pigs and rats, which pioneered the hypothesis that isocyanate skin exposure might promote airway inflammation/asthma [30-33]. The investigation also builds upon more recent mouse models of HDI and TDI asthma, which developed techniques for effectively delivering isocyanates (as mouse albumin conjugates) to the lower airways; thus overcoming technical challenges imposed by species difference between humans and mice ("scrubbing" action of nasal cavities and obligatory nasal breathing of mice), as well as respiratory tract irritation/toxicity by organic solvents (acetone, toluene) typically used for diluting isocyanate [15,22,31,34-37]. The findings of the present study are discussed in the context of disease (MDI asthma) pathogenesis and prevention.

## Materials and methods

### Reagents

Mouse and bovine albumin, triton X-100, sodium chloride, dithiothreitol (DTT), MDI, protease inhibitor cocktail and Tween 20 were from Sigma (St. Louis, MO). Urea and Tris-HCl were from American Bioanalytical (Natick, MA). Nonidet P40 substitute (Igepal CA-360) was from USB Corporation (Cleveland, OH). Acetone was from J.T. Baker (Phillipsburg, NJ). Ethylenediaminetetraacetic acid (EDTA) and phosphate buffered saline (PBS) were from Gibco (Grand Island, NY). Nunc Maxisorp™ microtiter plates were obtained through VWR International (Bridgeport, NJ). SuperSignal West Femto Maximum Sensitivity enhanced chemiluminescence substrate was obtained through Thermo Fisher Scientific (Rochester, NY). Tetramethylbenzidine (TMB) substrate was from BD Bioscience (San Jose, CA). Streptavidin conjugated alkaline phosphatase and p-nitrophenyl phosphate (pNPP) substrate were from Kirkegaard & Perry Laboratories (Gaithersburg, MD). Peroxidase conjugated rat anti-mouse anti-IgG_1_, and anti-IgG_2a _were from Pharmingen (San Diego, CA). Protein G Sepharose 4 Fast Flow was from GE Healthcare (Piscataway, NJ). Biotin-labeled rat anti-mouse IgE was from BioSource International, Inc. (Camarillo, CA). Imperial protein stain and rabbit anti-mouse IgG were from Pierce (Rockford, IL). Nitrocellulose and reducing gel electrophoresis buffer were from Bio-Rad (Hurcules, CA). Rabbit anti-tropomyosin, rabbit anti-collagen type 1/α2, and mouse anti-cytokeratin 14 were from Santa Cruz Biotechnology, Inc (Santa Cruz, CA).

### Animals and skin sensitization

**Figure 1:**
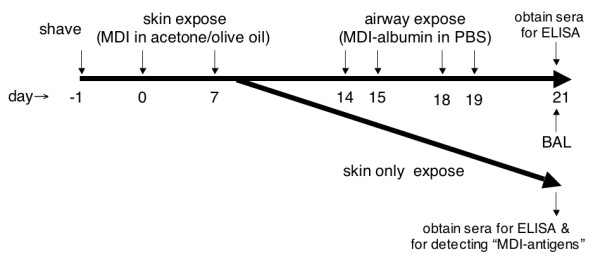
**Experimental time line**. The major time points of dermal and/or subsequent airway exposure as well as sample acquisition are depicted.

Female BALB/c mice, 9 to 12 weeks, from the National Cancer Institute (Frederick, MD), were used in all experiments. The backs of mice were shaved with electric clippers 1 day before exposure to 50 μl of MDI ranging in dose from 0.1%-10% weight/volume (w/v), delivered in a 4:1 acetone/olive oil "vehicle" (approximate surface area 0.5 - 1 cm^2 ^on right side). Control mice were identically exposed to 50 μL of an acetone/olive oil mixture without MDI. Mice were anesthetized during the skin exposure, and 20 minutes after application, the exposed area was cleansed with 70% ethanol. Mice were re-exposed a second time 7 days later on the opposite (left) side of their back. Serum of exposed mice was obtained on day 21 and analyzed by ELISA for MDI-specific antibodies, and used as a probe to detect MDI (exposure)-induced antigenic-modification of "self" mouse skin proteins. In some studies MDI skin exposed mice were subsequently exposed to MDI-albumin conjugates via the respiratory tract (see below). A time line of skin/airway exposures and sample acquisition is shown in Figure 1.

### Measurement of serum antibodies

Mouse sera samples were analyzed for MDI-specific antibodies using an enzyme-linked immunosorbant assay (ELISA), similar to that our laboratory has recently developed for measuring MDI-specific human antibodies [38]. Microtiter plates were coated with 1 μg/well of mouse albumin conjugated with MDI (see below), or control "mock exposed" mouse albumin, by overnight incubation at 4°C, in 0.1 M carbonate buffer (pH 9.5). Plates were "blocked" with 3% (w/v) bovine serum albumin before murine serum samples were titrated in blocking buffer. Sera were incubated for 1 hour at 25°C, followed by a 1:2000 dilution of peroxidase conjugated rat anti-mouse anti-IgG_1 _or anti-IgG_2a_. MDI-specific IgE was detected with biotin-labeled secondary rat anti-mouse IgE, followed by streptavidin-conjugated alkaline phosphatase. ELISAs were developed with TMB or p-NPP substrate and optical density (OD) measurements were obtained on a Benchmark microtiter plate reader from Bio-Rad. All samples were tested in triplicate to obtain average values expressed in figures.

MDI-specific IgG data are reported as end-titers; the reciprocal of the highest dilution that yields a positive OD reading, > 3 S.D. units above control serum from unexposed mice. Isocyanate-specific IgE data are represented as a binding ratio, as recommended in previous clinical studies, which is calculated as the (OD of wells coated with MDI-albumin) ÷ (OD of wells coated with control albumin) [39]. Total serum IgE levels were measured as previously described [40].

### MDI-albumin

MDI-mouse albumin conjugates used for ELISA and respiratory tract challenge were prepared under the reaction conditions recently defined to yield optimally antigenic MDI-conjugates with human albumin [38]. Mouse albumin in phosphates buffered saline (pH 7.2) at 5 mg/ml was mixed with a freshly prepared solution of 10% (w/v) MDI dissolved in acetone, to achieve a final MDI concentration of 0.1% (w/v). The reaction mixture was rotated end-over-end for 2 hours at room temperature, dialyzed against PBS and (0.2 μM) filtered. "Mock exposed" albumin was identically prepared, using only acetone (1% v/v final concentration) for the 2-hr exposure period. MDI conjugation to mouse albumin was verified based on characteristic shift in electrophoretic mobility, and absorbance at 250 nm, due to MDI's double ring structure [41]. In later experiments, for comparative purposes (with albumin purified from skin exposed to MDI in vivo, see below), we generated MDI-mouse albumin conjugates in vitro with varying levels of MDI/protein molecule, by varying the MDI concentration during conjugation reactions.

### Respiratory Tract Challenge with MDI-mouse albumin conjugates

Mice were lightly anesthetized with methoxyflurane and exposed to 50 μL of a 2 mg/ml solution of MDI-albumin or control "mock exposed" albumin in PBS by means of an intranasal droplet on days 14, 15, 18, and 19; and sacrificed by means of CO_2 _asphyxiation on day 21. Bronchoalveolar lavage (BAL) cell counts and differentials were performed as previously described [40].

### Processing of skin proteins

Mice were skin exposed to MDI or vehicle for 20 minutes, as described above; following which, the exposed area was wiped clean with 70% ethanol, surgically excised, and snap frozen in liquid nitrogen. Skin samples were then homogenized in a glass tissue grinder in an isotonic, pH buffered, detergent solution (20 mM Tris-HCl, 0.15 M NaCl, 1 mM EDTA, 1% Triton X-100, 0.5% Nonidet P40 and a cocktail of protease inhibitors). The homogenized samples were then microfuged at 16,000 x *g *for 5 minutes to obtain a "detergent soluble" fraction (supernatant) of skin proteins. Before Western blot analysis, detergent extracted skin samples were depleted of endogenous murine immunoglobulins by incubation with Protein G-coated sepharose beads, and clearance by centrifugation. The detergent insoluble fraction of skin samples was further homogenized in a strong denaturing buffer containing 9M urea and 50 mM DTT, to obtain a urea soluble fraction of skin proteins.

### Detection of antigenically modified skin proteins (MDI antigens)

Skin samples from MDI exposed mice were Western blotted with serum IgG from autologous mice that had been "skin-only" exposed to MDI, to detect "self" proteins antigenically modified by MDI exposure. Specificity controls included parallel blots with sera from mice exposed to vehicle only, and irrelevant (anti-ovalbumin) hyperimmune sera. Electrophoresis and Western blot were performed as previously described using pre-cast sodium dodecyl sulfate (SDS) acrylamide gels (4-15% gradient) from BioRad, and nitrocellulose membrane [42,43]. Nitrocellulose strips were incubated for 2 hrs with a 1:100 dilution of sera, washed extensively with PBS containing 0.05% Tween 20, incubated with a 1:2000 dilution of peroxidase conjugated anti-mouse IgG, and developed with enhanced chemiluminescence substrate.

### Purification of "MDI antigens" from exposed skin

Proteins from MDI exposed mouse skin were purified by a 2-step (isoelectric focusing/electroelution) process, guided by serum IgG from "skin only" exposed autologous mice, to detect antigenic modification. Preparative isoelectric focusing was performed using a Rotofor^® ^system from Bio-Rad, according to the manufacturers recommendations, to initially separate skin proteins into 20 fractions between pH 3 and 10, with subsequent re-focusing between pH 3 to 6, to increase resolution. Rotofor fractions containing proteins antigenically modified by MDI exposure were further fractionated and analyzed by parallel Western blot/SDS-PAGE, from which they were excised using a Bio-Rad Model 422 Electro-Eluter run at constant current (8-10 mA/glass tube) for 3-5 hrs. Purified proteins were aliquoted and further analyzed for protein sequence (see below) and confirmation of MDI-antigenicity via immunoblot with serum IgG from exposed mice.

### Protein identification

Liquid chromatography (LC) followed by tandem mass spectrometry (MS/MS) was performed by the Yale Keck Center on a Thermo Scientific LTQ-Orbitrap XL mass spectrometer, as previously described [44]. Briefly, purified proteins were reduced and carboxamidomethylated, trypsin digested and desalted with a C18 zip-tip column before MS/MS analysis. From uninterrupted MS/MS spectra, MASCOT compatible files (http://www.matrixscience.com/home.html) were generated, and searched against the NCBI non-redundant database [45,46]. For true positive protein identification, the 95% confidence level was set as a threshold within the MASCOT search engine (for protein hits based on randomness search). In addition, the following criteria must also have been met (1) two or more MS/MS spectra match the same protein entry in the database searched, (2) matched peptides were derived from trypsin digestion of the protein, (3) the peptides be murine in origin, and (4) the electrophoretic mobility must agree with the molecular weight. The identity of the purified proteins was further confirmed by Western blots with commercially available polyclonal or monoclonal antibodies (type I collagen, keratin-14, and tropomyosin), using hyperimmune anti-ovalbumin rabbit or mouse serum as a (negative) specificity control.

### Statistical analyses

Statistical significance was determined using ANOVA with a block design for pooled data from more than one experiment. Antibody data, calculated through 2-fold dilutions, were log(2) transformed for analysis.

## Results

### Skin exposure induces an MDI-specific antibody (i.e. systemic) response

**Figure 2:**
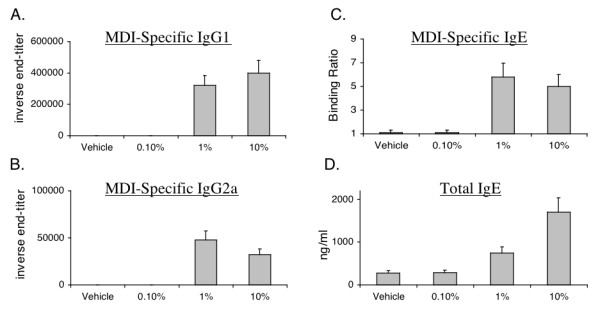
**Serum antibody responses to MDI skin exposure**. BALB/c mice were skin (only) exposed to vehicle (acetone/olive oil) or varying concentrations of MDI (0.1 - 10% w/v) as shown on X-axis. On day 21, serum levels of MDI-specific IgG_1_/IgG_2a _(inverse end-titer), IgE binding (ratio) and total IgE (ng/ml) were measured. Data shown are the mean ± SEM of 12 mice per group.

The capacity of MDI skin exposure to induce an MDI-specific antibody response was evaluated through ELISA analysis of sera from mice exposed to MDI diluted in acetone, at varying concentrations ranging from 0.1-10% weight/volume (w/v). We found that skin exposure to ≥ 1% MDI resulted in the development of high serum levels of MDI-specific antibodies. As shown in Figure 2, the end titers for MDI-specific antibody reached >1:100,000 and >1:30,000 for IgG_1 _and IgG_2a _subclasses respectively. MDI-specific IgE and total IgE serum levels were also elevated, up to 6-fold above control levels. The IgG and IgE induced by MDI skin exposure did not bind to unexposed proteins, or other reactive chemical "haptens" such as DNCB or adipoyl chloride (not shown).

### Influence of skin exposure on (secondary) respiratory tract exposure

**Figure 3:**
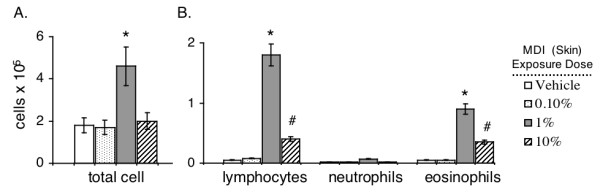
**Airway inflammatory responses to MDI in mice sensitized via skin exposure**. BALB/c mice that were initially skin exposed to vehicle or varying doses of MDI were subsequently exposed via the respiratory tract as described. On day 21, the number of cells recovered (by BAL) was determined. The data shown, are the mean ±SEM of 12 mice per group; *(*p *< .005) and ^#^*(p *< .05) compared to all other groups.

Mice initially exposed to MDI via the skin, were subsequently exposed via the respiratory, to a water soluble derivative of MDI (mouse albumin conjugates), in an adaptation of our murine HDI asthma model [22]. In the present experiments, mice that received only vehicle (acetone/olive oil) skin exposure, exhibited no change in bronchoalveolar lavage (BAL) cell numbers or differentials, when (airway) challenged with MDI-albumin conjugates. However, mice with previous (≥1%) MDI skin exposure developed significant airway inflammatory responses to respiratory challenge. The observed increase in total cell numbers of BAL samples (obtained 48 hours post exposure) was primarily due to increases in eosinophils and lymphocytes (Figure 3). Thus, respiratory tract exposure, to concentrations of MDI (albumin conjugates) that normally do not evoke cellular inflammation, causes pathologic changes (increased number of airway cells with Th2-profile) in mice previously exposed to MDI via the skin.

The initial MDI (skin) exposure dose was found to have a strong affect on the level of airway inflammation subsequently induced by respiratory tract challenge. The largest degree of airway inflammation was observed in mice initially (skin) exposed to MDI at a 1% (w/v) concentration, with more limited, albeit significant, inflammation in mice that had been skin exposed to 10% (w/v). The reason for the paradoxically limited airway inflammation in mice (skin) exposed to the highest test dose of MDI (10% w/v) remains unclear; however, analogous findings have been reported in HDI exposed mice [22]. A similar (non-linear dose-response) phenomenon is well-described for contact sensitization to many other reactive chemicals, e.g. formaldehyde, picryl chloride, DNCB [47].

### Respiratory tract exposure boosts serum levels of MDI-specific antibodies elicited by primary skin exposure

**Figure 4:**
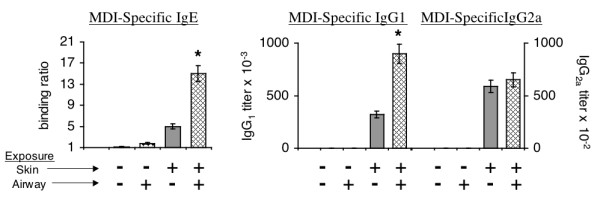
**Respiratory tract exposure boosts serum levels of MDI-specific antibodies elicited by primary skin exposure**. Serum levels of MDI-specific antibodies from mice (with (+) or without (-) prior skin exposure) following respiratory tract exposure to MDI albumin conjugates (+) or mock exposed albumin (-). Each bar represents the mean ± SEM for 12 mice; * *p *< .001 comparing skin exposed vs. skin + airway exposed

In mice with prior MDI skin exposure, subsequent respiratory tract exposure to MDI-albumin conjugates was found to boost MDI-immune sensitization, based on levels of MDI-specific serum IgG and IgE. As shown in Figure 4, statistically significant increases were detectable among Th2-associated subclasses/isotypes, IgG_1 _and IgE, but not in the Th1-associated subclass, IgG_2a_. Thus, in mice previously exposed to MDI via the skin, subsequent respiratory tract exposure to MDI (albumin conjugates) further boosts MDI immune sensitivity.

### Identification of MDI antigens in exposed skin

**Figure 5:**
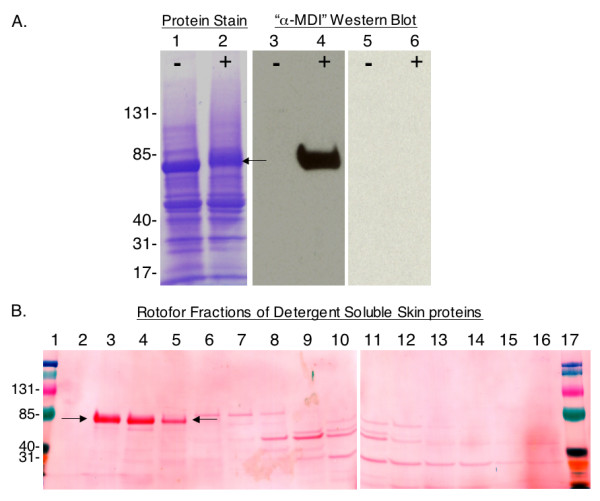
**Detection and fractionation of the major MDI antigen in detergent extracts of exposed skin**. (A) Proteins from (-) control or (+) 1% MDI exposed mouse skin, were separated by SDS-PAGE and stained with commassie blue or Western blotted with autologous sera from MDI skin exposed mice (lanes 3 and 4) or control mice (lanes 5 and 6). Arrow highlights major antigenic protein from exposed skin, with apparent shift in migration, indicating change in conformation/charge. (B) The MDI antigen, highlighted by arrows, was separated from other skin proteins by isoelectric focusing. Shown is Ponceau S protein staining of Rotofor^® ^fractions 2-16 after SDS-PAGE and transfer to nitrocellulose membrane. Lanes 1 and 17 contain prestained molecular weight markers.

**Figure 6:**
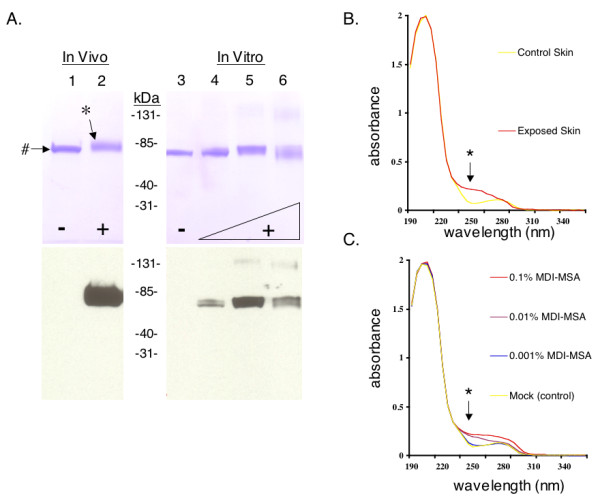
**Purification of antigenically modified albumin from in vivo exposed mouse skin**. (A) SDS-PAGE analysis (top) and Western blot with serum IgG from skin exposed mice (bottom) of the major MDI antigen (highlighted with *), purified from skin exposed *in vivo *to (+) 1% MDI and its corresponding protein purified from (-) control skin (highlighted with #). For comparison, MDI-albumin conjugates prepared in vitro using varying doses of MDI (0.001%, 0.01% and 0.1%, lanes 4 to 6 respectively) are shown to the right of the molecular weght markers. The MDI antigen was not recognized using control sera from vehicle expose mice or irrelevant hyperimmune mouse serum (not shown). (B) Ultraviolet light absorbance spectra of albumin purified from control or 1% MDI exposed skin. (C) For comparison, commercially purified mouse albumin and MDI-mouse serum albumin conjugates prepared in vitro were similarly analyzed. *Note increase in absorbance in the 250 nm range due to MDI's aromatic rings.

As shown in Figure 5A, detergent extracts from 1% MDI exposed skin contained a single antigenically-modified protein, specifically recognized by antibodies from autologous MDI skin (only) exposed mice, but not control mouse sera. The "MDI antigen" was purified from exposed skin by a 2-step process (Figure 5B, and 6A), and identified as albumin through LC-MS/MS analysis (see Additional file 1). The antigenically modified albumin from exposed skin exhibited biophysical properties consistent with MDI conjugation, when compared with albumin purified from vehicle-only exposed skin, or MDI-mouse albumin conjugates prepared in vitro; specifically, alterations in electrophoretic migration and change in absorbance at 250 nm (Figure 6A&6B).

**Figure 7:**
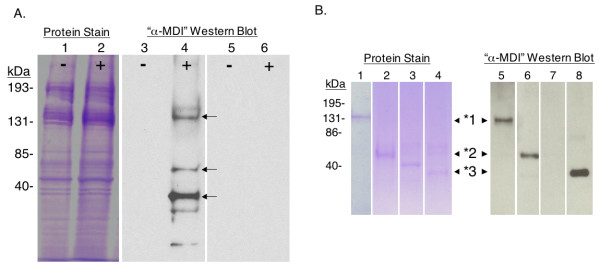
**Identification of MDI antigens in urea extracts of exposed skin**. (A) The detergent insoluble fraction of (-) control or (+) 10% MDI exposed skin tissue were further homogenized in 9 M urea, separated by SDS-PAGE, and stained for total proteins (lanes 1 and 2). Parallel Western blot with sera from autologous MDI skin exposed mice (lanes 3 and 4) vs. control mouse sera (lanes 5 and 6) identified at least three antigenically modified proteins (MDI antigens) in these samples; see arrows. (B) The MDI antigens from 10% MDI exposed mouse skin were purified and reanalyzed by protein stain following SDS-PAGE, and parallel Western blot with autologous sera from MDI skin exposed mice. Arrows highlight antigenically modified collagen (*1), keratin (*2) and tropomyosin (*3) from MDI exposed skin. Actin from unexposed mouse skin, which was not recognized by autologous sera, was run as a negative control (lane 3). MDI antigens were not detectable using control sera from vehicle expose mice or irrelevant hyperimmune mouse serum (not shown).

Additional "MDI antigens", specifically recognized by antibodies from MDI skin (only) exposed autologous mice, but not control mouse sera, were detectable in urea extracts from skin exposed to the highest test dose of MDI (10%), as shown (Figure 7A). Among these antigenically-modified proteins, the most prominent, based on recognition by serum IgG from skin exposed autologous mice, were purified through elecrophoretic fractionation methods, and identified by LC-MS/MS as pro-collagen type 1/α2, keratin 14, and tropomyosin (see Additional file 1). Their (MDI) antigenicity and identity were further confirmed by Western blot with autologous serum IgG from skin exposed mice (Figure 7B) and commercially available protein-specific (collagen, keratin, tropomysosin) antibodies (not shown).

## Discussion

In the present study, we utilized a murine MDI exposure model to demonstrate the capacity of skin exposure to induce immune sensitization to MDI, and promote airway inflammation upon subsequent respiratory tract exposure. The degree of secondary (respiratory tract) inflammation was found to depend upon the primary (skin) exposure dose, and exhibited a non-linear relationship that peaked when mice were skin exposed to 1% (w/v) MDI, and was paradoxically limited at 10-fold higher (skin) exposure doses; a phenomenon similar to that reported for HDI. Albumin in exposed skin was found to undergo antigenic as well as structural/conformational changes, consistent with MDI conjugation. Furthermore, MDI-mouse albumin conjugates were specifically recognized by serum IgE and IgG, and triggered heightened respiratory tract responses, in previously skin exposed mice. The data highlight mechanisms by which MDI skin exposure might contribute to the development of systemic immune sensitization and possibly MDI asthma.

The present findings are consistent with limited reports on MDI skin exposure in mice, despite differences in exposure protocols, and methods of assessing immunologic responses [48-51]. The findings are also consistent with data on the smaller, more volatile 6-carbon isocyanates, HDI and TDI, including, the non-linear "(skin) dose/(respiratory tract) response" and mixed Th1/Th2-like response to skin exposure [22,31,34,36,52]. Importantly, in all of these studies, the isocyanate concentrations found to induce immune responses via skin exposure (≤%1 w/v) were within the range commonly used in polyurethane production, and are likely experienced by workers in multiple occupational settings [8,28,53].

The presently described mouse model possesses distinct strengths as well as limitations compared with previously published animal studies of MDI and/or other isocyanate-induced asthma. One major strength is the use of skin as the primary exposure route for inducing a state of MDI-specific immune sensitization in which subsequent respiratory tract exposure leads to asthma-like inflammation. In this regard, the present investigation differs from prior studies attempting to model isocyanate-induced airway inflammation through "respiratory tract only" exposure, which have met limited success [15,31,49,54-60]. Another strength of the present study is the use of autologous serum IgG from skin exposed mice to identify immunologically-relevant protein targets for MDI conjugation and (antigenic) modification. The major weakness of the study, as viewed a priori, was the use of MDI-albumin conjugates, rather than MDI itself, for respiratory tract exposure (see Introduction for rationale), thus bypassing a major step between inhalation and inflammation. Retrospectively, however, the data suggest that albumin conjugates may be uniquely suited as antigens in modeling isocyanate asthma, especially secondary to initial skin exposure.

The data provide new insight into the reactivity of MDI with proteins present in the skin, which likely contributes to the development of MDI immune sensitization. At the 1% MDI exposure dose (which promoted the strongest secondary respiratory tract responses), only 1 skin protein, albumin, exhibited changes consistent with MDI conjugation (charge/conformation, ultraviolet light absorbance, antigenicity). Albumin is a major protein of the extracellular compartment of the skin, but has not been previously recognized as a target for isocyanate at that anatomical location [61]. However, albumin in airway fluid has been described as a major target for isocyanate conjugation in vivo following respiratory tract exposure [12-14,16,43,62]. Furthermore, albumin is the only known human protein whose conjugation with isocyanate confers specific recognition by human antibodies from exposed individuals [43,63]. Thus, the present data suggest that MDI conjugation to albumin in exposed skin creates an antigenic trigger that promotes subsequent airway inflammatory responses to respiratory tract exposure [22,35].

While albumin was the only MDI antigen detectable in skin exposed to 1% MDI, additional proteins were found to be antigenically-modified in skin samples exposed to the highest test dose (10%) of MDI. The significance of these proteins in response to MDI skin exposure will require further investigation. However, it is interesting to speculate the possibility that reactivity with MDI may alter their normal conformation in a manner that breaks "immune tolerance" given the reported association of anti-keratin antibodies with isocyanate asthma, and the pan-allergenicity of non-mammalian tropomyosin [64-66].

If the present data translate across species, they will provide important insight into pathogenic mechanisms of MDI asthma as well as practical guidance for disease prevention, among occupationally exposed individuals. The murine model will facilitate investigation of the role of specific genes, through transgenic technology, and provide a system for evaluating the effectiveness of different exposure interventions. The ELISA assay for MDI-specific IgG, described herein, may be helpful in assessing workplace skin exposure, which currently goes largely undetected, due to the lack of practical methodology for measuring. Furthermore, recognition of the ability to generate systemic immune sensitization to MDI via skin exposure, may promote increased awareness and use of personal (skin) protection, including gloves, overalls and head coverings.

## Conclusions

In summary, we developed a murine model to investigate the potential consequences of MDI skin exposure, which is relatively common in the numerous industries that utilize MDI to make polyurethane products. The present data demonstrate that MDI skin exposure can induce systemic immune sensitization and asthmatic-like inflammatory responses to subsequent respiratory tract exposure. Albumin was found to be a major target for MDI conjugation in exposed skin, and MDI-albumin conjugates were also shown to trigger heightened respiratory tract inflammation in previously skin exposed mice (vs. unexposed controls). The data may help explain the development of new MDI asthma cases despite extremely low workplace airborne MDI levels and provide practical guidance for exposure and disease prevention.

## Competing interests

The authors declare that they have no competing interests.

## Authors' contributions

AVW drafted the manuscript and supervised the in vitro immunology/biochemistry experiments. LX and ER performed in vivo skin and respiratory tract exposure studies, as well as BAL, and cell counts/differentials. JL performed the in vitro immunology/biochemistry experiments; ELISAs for MDI-specific IgG/IgE and total IgE, SDS-PAGE, Western blot, protein purification, and MDI-mouse albumin conjugate preparation. CAR organized the project and edited the manuscript. CAH conceived the original hypotheses underlying the overall project and supervised all aspects of the in vivo mouse studies. AVW, CAR, and CAH were together responsible for experiment design and data interpretation. All authors reviewed and approved the final manuscript.

## Supplementary Material

Additional file 1 **Antigenically modified proteins from exposed mouse skin identified by LC-MS/MS**. A table listing the positively identified peptides from the purified protein bands specifically recognized by serum IgG from MDI skin exposed mice. Click here for file
